# Exploring Perforated Jejunal GIST: A Rare Case Report and Review of Molecular and Clinical Literature

**DOI:** 10.3390/cimb46020076

**Published:** 2024-02-01

**Authors:** Milos Mirovic, Milica Dimitrijevic Stojanovic, Marina Jovanovic, Vesna Stankovic, Danijela Milosev, Natasa Zdravkovic, Bojan Milosevic, Aleksandar Cvetkovic, Marko Spasic, Berislav Vekic, Ivan Jovanovic, Bojana S. Stojanovic, Marko Petrovic, Ana Bogut, Miodrag Peulic, Bojan Stojanovic

**Affiliations:** 1 Department of General Surgery, Clinical Hospital Center Kotor, 85330 Kotor, Montenegro; mirovic.milos91@gmail.com; 2 Department of Pathology, Faculty of Medical Sciences, University of Kragujevac, 34000 Kragujevac, Serbia; milicadimitrijevic@medf.kg.ac.rs (M.D.S.); wesna.stankovic@gmail.com (V.S.); 3 Department of Pathology, University Clinical Center Kragujevac, 34000 Kragujevac, Serbia; danijelamilosevkg@gmail.com; 4 Department of Internal Medicine, Faculty of Medical Sciences, University of Kragujevac, 34000 Kragujevac, Serbia; marinna034@gmail.com (M.J.); natasasilvester@gmail.com (N.Z.); 5 Department of Surgery, Faculty of Medical Sciences, University of Kragujevac, 34000 Kragujevac, Serbia; draleksandarcvetkovic@gmail.com (A.C.); drmspasic@gmail.com (M.S.); vekicberislav@gmail.com (B.V.); miodrag.peulic@gmail.com (M.P.); bojan.stojanovic01@gmail.com (B.S.); 6 Center for Molecular Medicine and Stem Cell Research, Faculty of Medical Sciences, University of Kragujevac, 34000 Kragujevac, Serbia; ivanjovanovic77@gmail.com; 7 Department of Pathophysiology, Faculty of Medical Sciences, University of Kragujevac, 34000 Kragujevac, Serbia; bojana.stojanovic04@gmail.com; 8 City Medical Emergency Department, 11000 Belgrade, Serbia; bogutova12@gmail.com

**Keywords:** gastrointestinal stromal tumor, jejunal perforation, molecular pathology, CD117, surgical management, immunohistochemistry

## Abstract

This case report details a rare instance of a perforated jejunal gastrointestinal stromal tumor (GIST) in a 76-year-old female patient. The patient presented with acute abdominal pain and distension without any changes in bowel habits or episodes of nausea and vomiting. Initial diagnostics, including abdominal plain radiography and ultrasonography, were inconclusive; however, a computed tomography (CT) scan revealed pneumoperitoneum and an irregular fluid collection suggestive of small intestine perforations. Surgical intervention uncovered a 35 mm jejunal GIST with a 10 mm perforation. Histopathological examination confirmed a mixed cell type GIST with high malignancy potential, further substantiated by immunohistochemistry markers CD117, DOG1, and vimentin. Molecular analysis illuminated the role of key oncogenes, primarily KIT and PDGFRA mutations, emphasizing the importance of molecular diagnostics in GIST management. Despite the severity of the presentation, the patient’s postoperative recovery was favorable, highlighting the effectiveness of prompt surgical and multidisciplinary approaches in managing complex GIST cases.

## 1. Introduction

Gastrointestinal stromal tumors (GISTs) are the primary mesenchymal neoplasms of the gastrointestinal system, representing 1–3% of all gastrointestinal cancers [1,2,3]. Believed to originate from Cajal’s cells, crucial for regulating gastrointestinal motility, these tumors predominantly appear in the stomach (60–70%) and, to a lesser extent, in the small intestine (20–25%) [1,4,5]. They are less frequently found in areas like the colon, rectum, and esophagus [1,4]. Most GISTs are benign, but their clinical presentation can vary. The most common symptom is intestinal bleeding, occurring in about 40% of cases [6]. However, there are instances of critical emergencies, such as the rare jejunal GIST perforation leading to acute diffuse peritonitis [6].

GISTs are morphologically classified into spindle cell, epithelioid cell, and mixed cell types. Diagnosis relies heavily on histopathological examination, particularly the detection of CD117 protein [7]. The pathogenesis of GISTs involves crucial molecular pathways, with genetic mutations in oncogenes like KIT and PDGFRA playing a significant role in tumor growth and progression [8]. These insights are essential for targeted treatment approaches, which have notably improved outcomes in advanced cases [9]. Surgery is the cornerstone of treatment, offering a 5-year survival rate between 48–80% [10].

This case report highlights the diagnostic and management complexities of a rare perforated jejunal GIST presenting with acute diffuse peritonitis and underscores the importance of timely surgical intervention and a multidisciplinary treatment approach.

## 2. Case Presentation

A 76-year-old female patient with a notable medical history of urinary bladder carcinoma, arterial hypertension, coronary stent, and penicillin allergy presented to the emergency surgical unit at the University Clinical Center of Kragujevac. She reported acute onset abdominal pain and distension a few hours before admission. Notably, she had no changes in bowel habits or episodes of nausea or vomiting. On admission, her vital signs were stable: heart rate, blood pressure, respiratory rate, and body temperature within normal limits.

Physical examination revealed significant findings: abdominal distension, generalized tenderness, and guarding, indicating a potential acute abdominal pathology. Laboratory tests showed mixed results. Her hemoglobin level was 134 g/L, within the normal range of 110–157 g/L. The leukocyte count was slightly elevated at 7.9 × 10^9^/L (normal range: 3.7–10.0 × 10^9^/L) with a predominance of polymorphonuclear cells (79.10%). She exhibited hyperglycemia, with glucose levels at 10.0 mmol/L (normal: 3.8–6.1 mmol/L) and an increased C-reactive protein concentration of 59.5 mg/L (normal: 0.0–5.0 mg/L). Sodium levels were slightly decreased at 133 mmol/L (normal: 137–147 mmol/L). Renal function tests, including creatinine and blood urea nitrogen, along with pancreatic enzymes (amylase, lipase) and potassium levels, were within normal limits.

**Figure 1:**
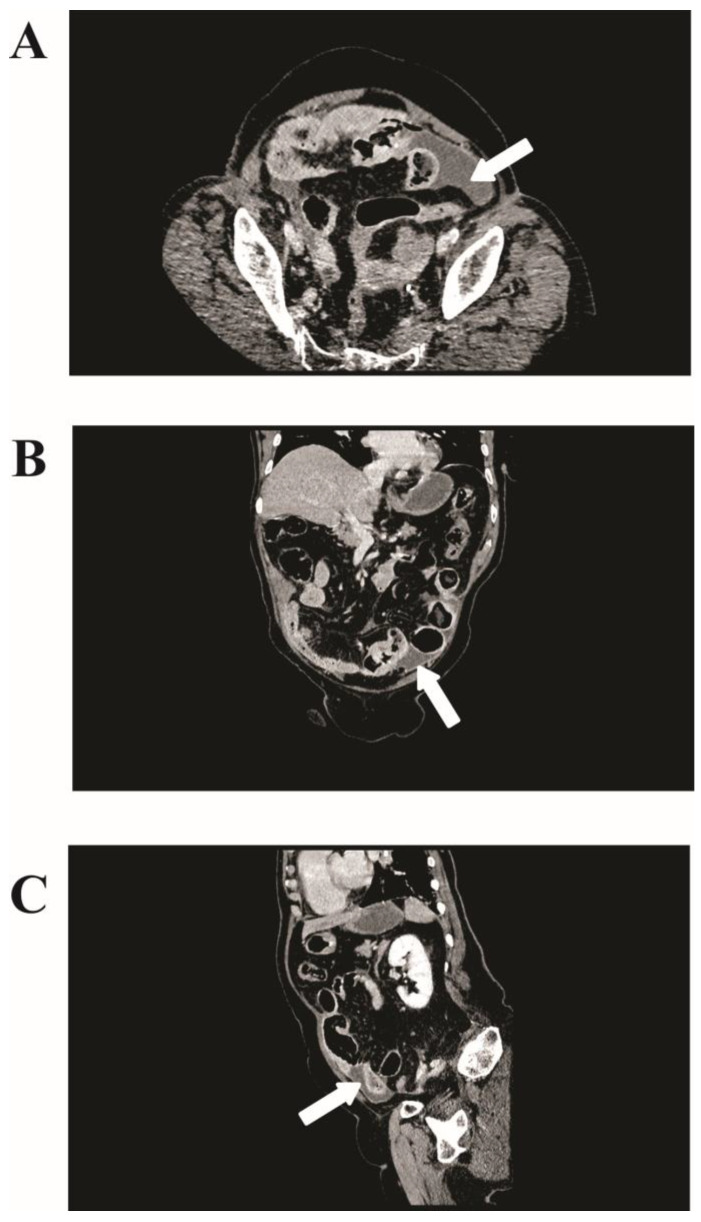
Computed tomography (CT) imaging of jejunal gastrointestinal stromal tumor (GIST). (**A**) Axial view from a contrast-enhanced CT scan showing an irregular fluid collection measuring 75 × 35 mm in the left lower quadrant of the abdomen (white arrows). (**B**) Coronal and (**C**) sagittal views further delineating the fluid collection’s positioning in the lower abdomen.

The initial imaging, comprising abdominal plain radiography and ultrasonography, was inconclusive, showing bowel distention but no clear signs of perforation. However, a crucial finding was noted in the computed tomography (CT) scan with intravenous contrast. It revealed pneumoperitoneum and a 75 × 35 mm irregular fluid collection at the pelvic inlet with air inclusions, suggestive of small intestine perforations (Figure 1).

The patient urgently underwent laparotomy, revealing acute diffuse peritonitis and significant contamination of the peritoneal cavity with purulent fluid and enteric content. A tumor was identified in the jejunum, 150 cm distal to the Treitz ligament, measuring 35 mm with a 10 mm perforation. Segmental jejunal resection, including the tumor, with clear macroscopic margins, was performed, followed by side-to-side handsewn small intestine anastomosis and irrigation drainage. Post-operatively, her gastrointestinal motility and oral intake normalized, although she developed basal bilateral pneumonia on the fifth postoperative day, which was effectively managed with antibiotic therapy.

**Figure 2:**
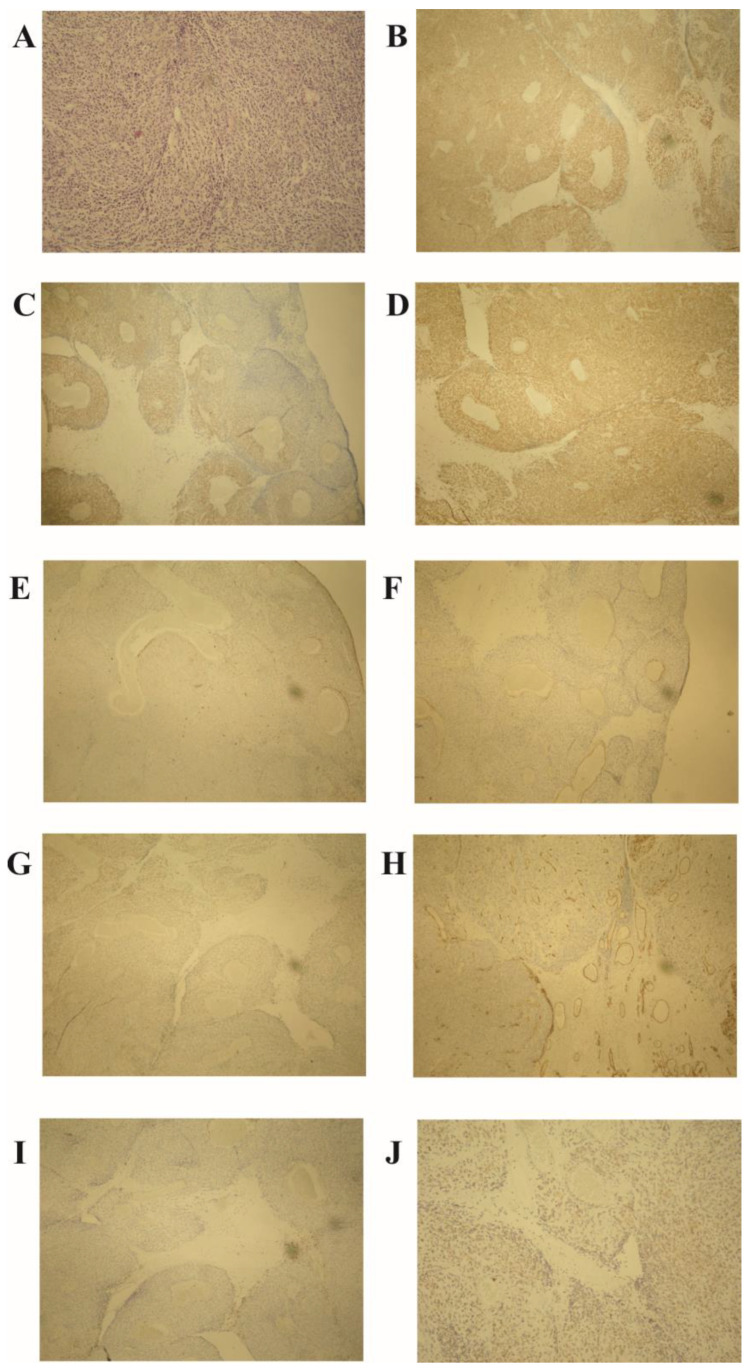
Histopathological analysis of the jejunal gastrointestinal stromal tumor (GIST). (**A**) High mitotic activity (over 5 per 50 HPF) and palisaded-vacuolated morphology in spindle tumor cells (hematoxylin–eosin stain, magnification ×100). Immunohistochemistry demonstrates positivity for (**B**) CD117, (**C**) DOG1, and (**D**) vimentin, with negativity for (**E**) S-100 protein, (**F**) α-smooth muscle actin (SMA), (**G**) CD99, (**H**) CD34, and (**I**) LCA. (**J**) Ki-67 proliferation index at 25% (magnification ×40). Note: HPF—high-power field; CD—cluster of differentiation; DOG1—discovered on GIST-1; SMA—smooth muscle actin; LCA—leukocyte common antigen; Ki-67—a marker for cell proliferation.

Histopathological examination of the excised specimen revealed a jejunal GIST with mixed cell type, predominantly spindle cell morphology, and partly palisaded-vacuolated morphology, showing transmural infiltration of the jejunum and a high malignancy potential, evidenced by a mitotic rate of more than 5 per 50 high-power fields (HPF) (Figure 2A). The tumor measured 35 mm. Immunohistochemical analysis showed positivity for CD117 (Cluster of Differentiation 117), DOG1 (Discovered on GIST-1), and vimentin, and negativity for S100 protein, SMA (smooth muscle actin), CD99 (Cluster of Differentiation 99), CD34 (Cluster of Differentiation 34), and LCA (leukocyte common antigen) (Figure 2B–I). The Ki-67 proliferation index was approximately 25% (Figure 2J). Seven lymph nodes were harvested, none of which showed tumor involvement. Surgical margins were clear, without lymphovascular or perineural invasion. The tumor was classified as pT2N0M0 per the AJCC TNM classification and staged as IIIb, given its size and high mitotic rate.

Subsequently, her case was reviewed at a Multidisciplinary Team Meeting. It was decided that the patient would follow a surveillance plan without adjuvant treatment. Two years into this follow-up period, she remained free of any signs of recurrence or systemic dissemination of the disease, demonstrating a favorable outcome under the watchful waiting approach.

## 3. Discussion

Gastrointestinal stromal tumors (GISTs) represent the most prevalent mesenchymal neoplasms within the gastrointestinal tract in adults, especially in those over 40 years of age, with a peak incidence between 60 and 65 years [11]. There is a slightly higher prevalence in males compared to females, and this trend appears consistent across various geographic and ethnic groups [4]. GISTs most commonly arise in the stomach (60–70%), followed by the small intestine (25–35%), and are least frequently found in the jejunum (10%) [12].

The case of a perforated GIST in the jejunum, as presented in this instance, is notably rare. Such cases have been sporadically reported in the English medical literature, underscoring their unusual occurrence (Table 1) [4,6,12,13,14,15,16,17,18,19].

Understanding the molecular basis of GISTs is crucial, as these neoplasms are driven by specific genetic mutations and alterations in signaling pathways [20]. The interplay of these molecular factors not only influences the tumor’s location and development but also its clinical behavior, including rare presentations like perforation.

### 3.1. Genetic Basis and Molecular Pathogenesis of Gastrointestinal Stromal Tumors (GISTs)

Gastrointestinal stromal tumors are primarily believed to originate from the digestive system’s pacemaker cells, known as Cajal cells, which are found from the esophagus to the rectum [21]. Recent findings, however, have expanded this understanding, suggesting that GISTs can also develop from telocytes or smooth muscle cells [8]. These tumors represent a heterogeneous group, with molecular subtypes largely defined by activating mutations. The most common mutations are found in the KIT proto-oncogene (KIT) or the platelet-derived growth factor receptor alpha (*PDGFRA*) gene [22].

About 5% of GISTs are categorized as syndromic, linked to hereditary mutations in genes such as *KIT*, *PDGFRA*, *neurofibromin*, and succinate dehydrogenase B/C/D (*SDHB/C/D*)—associated with Carney Stratakis syndrome [23]. There is also a non-hereditary form known as the Carney triad syndrome, characterized by the epigenetic silencing of the SDHC gene [24]. For patients with GISTs showing a neurofibromin 1 (*NF1*) mutation or a deficiency in the succinate dehydrogenase (SDH) complex, genetic counseling is strongly recommended [25].

The implementation of next-generation sequencing (NGS) has significantly enhanced our understanding of GISTs [26]. Historically, 85–90% of GISTs were identified with activating mutations in the KIT or PDGFRA genes. However, the molecular mechanisms in the remaining 10–15%, known as historical wild-type (WT) GISTs, were unclear [27]. Through NGS, it has been revealed that mutations in *KIT/PDGFRA* are also common in historical WT GISTs. Consequently, these mutations are now recognized as the primary oncogenic drivers in approximately 92–93% of all GIST cases [8,28]. In about 5–7.5% of all GISTs, the driving oncogenic mechanism is linked to a deficiency in the SDH complex [7]. In cases where neither *KIT/PDGFRA* mutations nor SDH complex deficiencies are detected, other rare driver alterations have been identified, including changes in the rat sarcoma virus (*RAS*) gene family, the *v-Raf* murine sarcoma viral oncogene homolog B1 (*BRAF*) gene, *NF1*, the neurotropic tyrosine receptor kinase 1–3 (*NTRK1–3*) genes, and the fibroblast growth factor receptor 1–4 (*FGFR1–4*) genes [29,30]. Despite these advancements, “true” WT GISTs, which lack any identifiable driving alterations even after comprehensive molecular analysis, remain extremely rare [31].

#### 3.1.1. The Role of KIT Mutations in GIST

The *KIT* gene plays a pivotal role in the development of GISTs [31]. It encodes the 145 kDa receptor tyrosine kinase c-KIT, which is a part of the type III receptor tyrosine kinase family [32]. This group includes other significant receptors like the platelet-derived growth factor receptors A and B (PDGFRA, PDGFRB), the macrophage colony-stimulating factor receptor (CSF1R), and the FL cytokine receptor (FLT3) [31]. KIT is composed of several domains: an extracellular domain, a juxtamembrane domain, and two tyrosine kinase domains. The kinase domain of KIT is maintained in an inactive state through auto-inhibition under normal circumstances [32].

The activation of KIT is intricately tied to its ligand, the stem cell factor (SCF) [33]. The binding of SCF to KIT initiates a cascade of molecular events, including enzyme dimerization and ATP binding, which results in auto phosphorylation [34]. This process activates several downstream pathways, notably the mitogen-activated protein (MAP) kinase cascade and the phosphoinositide 3-kinase/protein kinase B (PI3K/AKT) pathway. These pathways play a vital role in regulating various cellular processes. This includes the transcriptional regulation of genes such as MYC (myelocytomatosis viral oncogene), ELK (ETS-like gene), CREB (cAMP responsive element binding protein), and FOS (FBJ murine osteosarcoma viral oncogene homolog), along with fostering anti-apoptotic effects in cells [32].

KIT mutations play a critical role in the oncogenesis of GISTs. Approximately 70% to 80% of GISTs exhibit mutations in KIT [35]. These mutations result in the autonomous activity of the KIT protein, independent of SCF binding. This aberrant activity leads to the activation of multiple downstream signals such as MAPK (mitogen-activated protein kinase), AKT, S6K (ribosomal protein S6 kinase), STAT1 (signal transducer and activator of transcription 1), and STAT3 (signal transducer and activator of transcription 3), all of which contribute to the development and progression of GISTs [36].

An important factor in the progression of GISTs is the interaction between KIT and the ETS (erythroblast transformation-specific) family member, ETV1 (ETS variant transcription factor 1) [37]. ETV1, highly expressed in GISTs, acts as a transcriptional master regulator and forms a positive feedback loop with KIT, which is crucial for GIST growth [37]. The combination treatment targeting both KIT and downstream pathways has been shown to be effective in suppressing GIST growth both in vivo and in vitro [38].

#### 3.1.2. PDGFRA Mutations in Gastrointestinal Stromal Tumors

The proto-oncogene *PDGFRA*, located on chromosome 4 (q12), plays a significant role in the development of GISTs [39]. Like *KIT*, *PDGFRA* encodes a class III receptor tyrosine kinase (RTK) and is structurally homologous to KIT RTK. In GISTs, *PDGFRA* mutations are less frequent than *KIT* mutations but are still present in approximately 10% to 15% of cases [40]. These mutations result in constitutive activation of PDGFRA, independent of ligand binding, and trigger downstream signaling pathways similar to those activated by KIT mutations. Predominantly, these pathways include RAS (rat sarcoma virus)/RAF (rapidly accelerated fibrosarcoma)/MAPK and PI3K/AKT signaling [40].

GISTs with *PDGFRA* mutations often exhibit distinct characteristics: they are typically located in the stomach, present an epithelioid morphology, and follow an indolent clinical course [41]. Among the various *PDGFRA* mutations, the most prevalent is the *p.D842V* mutation, found in 60% to 65% of *PDGFRA* mutant GISTs and accounting for about 5% of all GISTs [42,43]. This mutation leads to a stable conformational structure of the tyrosine kinase in its active form [43].

In GISTs, the majority of *PDGFRA* mutations affect exon 18, which encodes the activation loop of the intracellular (IC) domain [39]. These mutations are present in up to 15% of all GISTs. Less frequently, mutations can be found in exon 12, affecting the juxtamembrane domain (JMD), and in exon 14, which encodes the ATP binding site of the tyrosine kinase domain (TKD) [44,45]. These latter mutations are relatively rare, occurring in up to 2% and 1% of all GISTs, respectively [46]. The exon *18 D842V* mutation, in particular, is significant due to its prevalence and its impact on tyrosine kinase activity in GISTs [43].

#### 3.1.3. SDH Deficiency in GISTs without KIT/PDGFRA Mutations

In gastrointestinal stromal tumors that lack KIT or PDGFRA mutations, a frequent molecular alteration is a deficiency in the succinate dehydrogenase (SDH) complex [31]. This complex, crucial in the Krebs cycle and respiratory chain, comprises four subunits encoded by tumor suppressor genes: *SDHA* on chromosome 5, *SDHB* on chromosome 1, *SDHC* on chromosome 1, and *SDHD* on chromosome 11 [47].

The functional loss in these mitochondrial enzymes leads to the accumulation of succinate, inhibiting dioxygenases, including ten–eleven translocation methylcytosine dioxygenases (TETs) and histone lysine demethylases (KDMs) [48]. This disruption allows hypoxia-inducible factor 1a (HIF-1a) to accumulate, upregulating the transcription of genes like IGF1R and VEGFR. Such molecular changes, combined with DNA hypermethylation, are implicated in the malignant transformation of interstitial Cajal cells into GISTs [49].

SDH-deficient GISTs, accounting for about 5–7.5% of all GIST patients, are often identified by the absence of immunohistochemical staining for SDHB [8]. Complex mutations in SDH genes, typically associated with germline mutations, are common in these GISTs [50]. About half of these patients possess germline-inactivating mutations in an SDH gene, often leading to syndromic diseases [51]. Notable among these are the Carney–Stratakis syndrome, characterized by gastric GISTs and paragangliomas, and the Carney triad syndrome, involving GISTs, paragangliomas, and lung chondromas, often linked to epigenetic silencing of *SDHC*. Both these syndromes, along with Leigh syndrome, a neurodegenerative disorder, are associated with SDH deficiencies in GISTs [24,52].

The immunohistochemical profile of SDH-deficient GISTs also reveals significant clinical insights. For example, patients with *SDHA*-positive GISTs tend to be older, predominantly female, and show a higher rate of liver metastasis compared to those with *SDHA*-negative GISTs. Interestingly, the mitosis rate, tumor size, and overall clinical course appear similar between SDHA-positive and -negative cases [53].

#### 3.1.4. RAS and BRAF Mutations in Gastrointestinal Stromal Tumors

Mutations in the *RAS* family genes and *BRAF*, though infrequent, occur in GISTs [54]. RAS proteins function as molecular switches, alternating between active guanosine triphosphate (GTP)-bound and inactive guanosine diphosphate (GDP)-bound states. *KRAS* (Kirsten rat sarcoma viral oncogene homolog), a significant member of this family, is frequently mutated in various cancers, including pancreatic, colorectal, and lung cancers [55]. *KRAS* mutations in GISTs are rare, identified in only 5% of cases, predominantly at codons 12 or 13 [54]. These mutations, which may hinder inactivation by GAPs (GTPase-activating proteins), can be primary or secondary, the latter often emerging post-imatinib treatment in KIT/PDGFRA mutant GISTs [56].

The *BRAF* proto-oncogene, located on chromosome 7, encodes a serine–threonine kinase pivotal for the MAPK signaling pathway [57]. *BRAF* mutations are categorized into three classes: class one mutations result in a constitutively active monomer, class two mutations lead to an active dimer, and class three mutations reduce or abolish kinase activity [57]. The most clinically relevant mutation, *BRAF V600E*, found in various cancers, is a rare event in GISTs, occurring in less than 1% of adult patients [58]. However, the *BRAF V600E* mutation is notable in GISTs with wild-type KIT/PDGFRA, representing an early tumorigenic event [59]. Huss et al. reported *BRAF* mutations in about 1.6% of all GISTs and 3.9% of wild-type GISTs, indicating its significance in GIST development [60].

#### 3.1.5. Diverse Genetic Alterations beyond KIT and PDGFRA in GISTs

Recent research has expanded the understanding of genetic alterations in GISTs beyond the well-known mutations in *KIT* and *PDGFRA*. *EGFR* mutations, found in a small fraction (0.93%) of primary GISTs, are associated with gastric location, female gender, and a low recurrence rate. Notably, these *EGFR* mutations do not overlap with mutations in *KIT*, *PDGFRA*, *KRAS*, or *BRAF* [61]. Additionally, a *PIK3CA* mutation was reported in a GIST case with a concurrent KIT exon 11 deletion [62].

In wild-type GISTs lacking mutations in *KIT, PDGFRA, RAS* signaling genes, or *SDH* deficiency, a study identified mutations in several other genes, including *ARID1B* (AT-rich interaction domain 1B), *ATR* (Ataxia Telangiectasia and Rad3 related), *FGFR1* (fibroblast growth factor receptor 1), LTK (leukocyte receptor tyrosine Kinase), SUFU (suppressor of fused homolog), *PARK2* (Parkin RBR E3 ubiquitin protein ligase), and *ZNF217* (zinc finger protein 217). Particularly noteworthy are *FGFR1* gene fusions and an *ETV6-NTRK3* (ETS variant 6-neurotrophic receptor tyrosine kinase 3) fusion found in this subgroup [63,64]. The latter fusion, also observed in breast carcinoma, comprises the helix-loop-helix dimerization domain of *ETV6* fused to the protein tyrosine kinase domain of NTRK3 [64,65].

The *NF1* gene, a large tumor suppressor gene located on chromosome 17, encodes neurofibromin, which is involved in the RAS/MEK/MAPK and mTOR pathways [66]. Inactivating *NF1* mutations can lead to neurofibromatosis type 1 (NF1), an autosomal dominant disorder predisposing to cancer development [66]. Different inactivating mutations in *NF1* result in varied clinical presentations and are linked to other modifier genes contributing to the pathogenesis [67]. In GISTs, *NF1* mutations are relatively rare, constituting about 1–2.4% of all GIST cases [46]. NF1-associated GISTs often exhibit immunohistochemical expression of *KIT, DOG1*, and *SDHB*, along with loss of heterozygosity at 14q and 22q, occasionally accompanied by *KIT* mutations or alterations in the notch signaling pathway [68,69].

Alterations in the protein phosphatase 2 regulatory subunit A alpha (*PPP2R1A*) can impair the function of protein phosphatase 2A (*PP2A*). A study found *PPP2R1A* mutations in 18% of GISTs, with most of these cases also harboring mutations in *KIT, PDGFRA*, or *RAS* family genes or showing *SDH* deficiency [70]. Additionally, a potential link between *BRCA2* (breast cancer 2, early onset) mutations and GIST development has been reported, with an individual carrying a *BRCA2* mutation developing prostate cancer, breast cancer, and GIST [71].

### 3.2. Clinical Symptomatology of Gastrointestinal Stromal Tumors

Gastrointestinal stromal tumors typically develop within the walls of the stomach or small intestine and often grow into the empty space between the abdominal organs [72]. Consequently, many GISTs may not cause symptoms immediately unless they reach a significant size or are located in specific areas [72]. Smaller GISTs, in particular, might remain asymptomatic and are often discovered incidentally during evaluations for other medical issues. These smaller tumors usually exhibit slow growth [5].

A notable symptom associated with GISTs is gastrointestinal bleeding, which is a common consequence of the fragile nature of these tumors. The manifestation of this bleeding varies depending on the speed of blood loss and the tumor’s location. Rapid bleeding can lead to vomiting of blood, which may resemble coffee grounds when partially digested. Brisk bleeding into the stomach or small intestine can result in black and tarry stools while bleeding into the large intestine might cause the stool to appear red with visible blood. In cases of slow bleeding, symptoms might not be immediately apparent, but over time, it can lead to anemia characterized by fatigue and weakness [1].

Other symptoms of GISTs include abdominal pain, a noticeable mass or swelling in the abdomen, nausea and vomiting, early satiety, loss of appetite, and weight loss. In cases where the tumor grows large enough to obstruct the gastrointestinal tract, patients may experience severe abdominal pain and vomiting due to the blockage of food passage [73].

### 3.3. Perforation of Jejunal Gastrointestinal Stromal Tumors

Jejunal GISTs are unique in their clinical presentation and prognosis due to their rarity and tendency for severe complications. While GISTs are relatively uncommon mesenchymal neoplasms of the gastrointestinal tract, those originating in the jejunum are especially rare [74]. Perforation in jejunal GISTs is an infrequent but serious complication, leading to acute diffuse peritonitis [12]. The perforation of GISTs, particularly in the jejunum, is most often spontaneous and associated with a poor prognosis [1]. These ruptures typically occur in the stomach and small bowel, with the majority happening without any preceding trauma or clear precipitating factor [1].

There are three types of GIST rupture described in the literature: closed perforation (abscess type), hemoperitoneum leading to rupture of the hematoma capsule in the tumor (hemoperitoneum type), and free perforation (bowel perforation type) [12]. The latter, which includes cases like the presented jejunal GIST perforation, is the rarest and may result from obstruction with increasing intraluminal pressure or tumor erosion, leading to mural necrosis [1,75].

### 3.4. Diagnosis of Gastrointestinal Stromal Tumors

Diagnosing GISTs can be challenging, as no single diagnostic procedure guarantees 100% accuracy [76]. Commonly utilized examinations include barium studies of the gastrointestinal system, computed tomography (CT), and angiography. However, these methods cannot definitively diagnose GISTs on their own [76]. Magnetic resonance imaging (MRI) is noted to provide superior information compared to CT in the preoperative assessment of these tumors. Significantly, about one-third of GISTs are discovered incidentally, often during investigations for other medical conditions or symptoms [1]. This highlights the importance of considering GISTs in the differential diagnosis when imaging reveals unexpected abdominal masses or anomalies, as was the case in our report of a jejunal GIST presenting with acute symptoms.

### 3.5. Histopathology of Gastrointestinal Stromal Tumors

The histopathological diagnosis of gastrointestinal stromal tumors (GISTs) relies significantly on the morphological characteristics of tumor cells and immunohistochemical markers. GISTs are primarily classified into three morphological types: spindle cell type (70%), epithelioid cell type (20%), and mixed cell type (10%). These morphological variations reflect the diverse cellular origins and biological behaviors of GISTs [4,77].

Immunohistochemistry plays a pivotal role in the diagnosis of GISTs. Most GISTs are positive for c-kit (CD117) and DOG1, with 60–70% of cases also expressing CD34 [4,77]. Additionally, 30–40% of GISTs show positivity for Smooth Muscle Actin (SMA), 10% for vimentin, and 5% for S100 protein [78]. These markers aid in distinguishing GISTs from other mesenchymal tumors of the gastrointestinal tract. In our case presentation, all these markers were tested to confirm the diagnosis and understand the tumor’s molecular profile better.

### 3.6. Prognosis of Gastrointestinal Stromal Tumors

The prognosis of GISTs is influenced by several factors, primarily the stage of cancer, which is determined through physical exams and tests [79]. Stages range from I through IV, with lower stages indicating less spread of cancer. Stage IV signifies more extensive spread. The stage of cancer plays a crucial role in determining treatment strategies and survival statistics.

Additional factors impacting the prognosis include the tumor’s location, size, and mitotic rate, as well as whether the tumor has ruptured. High mitotic activity (more than 5 mitoses per 50 high-power fields) and larger tumor size (more than 5 cm) are indicative of higher malignant potential and poorer prognosis. The Ki67 index, particularly when it exceeds 22%, is also a strong predictor of poor survival [79]. The presence of mutations in the KIT or PDGFRA genes, common drivers of GIST growth, can affect the tumor’s response to targeted therapies, influencing prognosis and treatment options [80].

In cases where GISTs lead to perforation, as in our case of jejunal GIST, the prognosis becomes more dismal. Perforated GISTs significantly lower the five-year survival rate, likely due to peritoneal dissemination of tumor cells. This dissemination significantly lowers the five-year survival rate to around 24%. Thus, early detection and intervention are critical in managing GISTs and improving patient outcomes [1].

### 3.7. Treatment of Gastrointestinal Stromal Tumors

The treatment of GISTs has evolved significantly over recent years. The mainstay of treatment for localized GISTs is surgical resection, which offers the only potentially curative option [81,82]. Approximately 85% of these tumors can be completely resected, though the incidence of recurrence and metastasis post-radical surgery stands at about 50% [83]. Achieving a negative margin is crucial to prevent local recurrence, and lymphadenectomy is typically not indicated due to the rarity of lymph node involvement [4].

In addition to surgery, targeted therapy plays a crucial role, especially for advanced GISTs or in cases where surgery is not feasible [84]. Tyrosine kinase inhibitors (TKIs) like imatinib and sunitinib are used to block signals essential for tumor growth. These therapies have significantly improved overall survival rates and reduced recurrence after surgery [85].

The ESMO–EURACAN guidelines for treating Gastrointestinal Stromal Tumors prioritize surgical intervention as the primary treatment for localized cases, with complete surgical excision of the tumor being the standard approach. In instances where laparoscopic surgery is considered, it must adhere to the strict principles of oncological surgery. However, this method is not recommended for larger tumors due to the increased risk of tumor rupture and subsequent high relapse risk. In situations where achieving a complete removal (R0 surgery) might lead to significant functional loss, a marginally less thorough surgery might be acceptable, especially in low-risk tumors where this approach does not significantly impact overall survival. For patients who have previously undergone an incomplete excision, considering a second surgery is a viable option, depending on the feasibility and the expected functional outcomes [86].

Regarding post-surgical treatment, a three-year course of imatinib is recommended for those at high risk of cancer recurrence. This treatment does not apply to GISTs with specific genetic mutations (PDGFRA D842V), which show resistance to this therapy. In certain cases, a higher dose of imatinib is advised, especially for tumors with exon 9 KIT mutations. Adjuvant treatment is generally not recommended for specific GIST subtypes due to their natural behavior and resistance to typical treatments. In the unique and challenging situation of pediatric GISTs, international collaboration is necessary to establish effective treatment protocols. Additionally, in situations where tumors have ruptured during surgery, leading to the potential spread of cancer cells, imatinib therapy is strongly advised to mitigate the risk of relapse. In cases where a less invasive surgery might be possible after reducing the tumor size with imatinib, this approach is considered standard, especially when significant surgical procedures, such as total gastrectomy, are involved. Before initiating this therapy, a biopsy is recommended to confirm the diagnosis and identify any resistant genotypes, adapting the treatment plan accordingly. Surgery is then conducted following the maximal response to the treatment, usually within 6 to 12 months [86].

Additionally, the 2023 GEIS Guidelines for GISTs outline a comprehensive approach to the management of localized GISTs. Surgical removal of the tumor is the preferred method of treatment, aiming for a complete excision with a margin of at least 1 cm, ensuring that the pseudocapsule remains intact. For tumors that are less aggressive or not visibly affecting lymph nodes, extensive lymph node removal is not necessary. The guidelines suggest a conservative surgical approach that spares healthy tissue. In cases where the tumor is unresectable due to the involvement of major arteries, a multidisciplinary team should assess the potential for a more extensive surgery. However, such extensive procedures are generally discouraged. Laparoscopic surgery is another option, but it is typically not recommended for larger tumors (over 10 cm) due to the risk of tumor rupture [87].

In terms of surgical outcomes, a microscopic incomplete resection (R1) does not necessarily correlate with a higher recurrence risk or decreased survival, unlike a macroscopic incomplete resection (R2), which has a poorer prognosis. The decision to perform a second surgery after an R1 resection depends on the potential risks and outcomes. For smaller GISTs (less than 2 cm), endoscopic management is often preferred, with regular monitoring recommended if biopsy is not feasible. In cases of tumor rupture, immediate treatment with imatinib is advised due to the high risk of relapse [87].

Adjuvant therapy with imatinib, particularly for high-risk patients, has been shown to improve survival rates and reduce recurrence. Current debates in the medical community focus on the optimal duration of adjuvant imatinib treatment, with ongoing studies comparing the effects of 3 versus 5 years of therapy. Additionally, the guidelines recommend considering adjuvant imatinib in specific genetic mutations of GISTs. In the context of neoadjuvant treatment, imatinib is considered for locally advanced GISTs to facilitate subsequent surgical procedures, with a typical treatment duration of 6 to 12 months before surgery. The overall management strategy for localized GISTs includes a comprehensive evaluation of the tumor’s molecular profile before initiating adjuvant imatinib and a multidisciplinary approach for advanced cases where pre-surgical imatinib treatment may be beneficial [87].

### 3.8. Current Frontiers in Gastrointestinal Stromal Tumor Research: A State-of-the-Art Overview

Recent research in the field of GISTs has focused on several key areas, enhancing our understanding and management of these tumors.

Molecular Subtypes and Genomic Studies: There’s been significant progress in comprehensively understanding the molecular mechanisms, especially in GISTs that lack KIT or PDGFRA mutations. Detailed characterization of the molecular underpinnings, particularly SDH deficiency in GIST, has been a focus. This has implications for the clinical responses of these GISTs to conventional tyrosine kinase inhibitors [88].

Mutation-Specific Treatments and Resistance Patterns: The relationship between specific mutations within GISTs and the tumor’s behavior, including drug sensitivity, is a major area of research. For instance, the presence of certain mutations in KIT exons and their correlation with the effectiveness of specific TKIs has been extensively studied. This includes the exploration of resistance mutations and their impact on treatment strategies [89].

Palliative and Supportive Care: Clinical trials are actively seeking better methods to reduce symptoms and side effects of current GIST treatments, aiming to improve patient comfort and quality of life [90].

Targeted Therapy Drugs: The recent advancements in genetics have significantly influenced the treatment of GISTs, leading to the development of targeted drugs like imatinib, sunitinib, regorafenib, and ripretinib. These drugs are formulated to act on cells with specific genetic mutations associated with GISTs and have shown effectiveness in treatment. However, their long-term impact and the most effective treatment protocols, including the duration of therapy, are subjects of ongoing clinical trials. Concurrently, the field of immunotherapy for soft tissue sarcomas, such as GISTs, is rapidly advancing. It encompasses a range of potential treatments, including cytokine-based therapy, immune checkpoint inhibitors, anti-KIT monoclonal antibodies, bi-specific monoclonal antibodies, and cell-based therapies. This multifaceted approach to immunotherapy is currently under comprehensive review and research, aiming to establish new standards of care in the treatment of GIST [8,91].

Emerging Treatments: The treatment of PDGFRA exon 18 mutated GISTs with avapritinib is being explored. There’s also interest in understanding the broader spectrum of challenges and opportunities in GIST treatment as the field enters a new decade of discovery and innovation [92].

Overall, the landscape of GIST research is rapidly evolving, with a strong emphasis on personalized medicine based on molecular profiling and genetic understanding of the tumors. This approach is expected to lead to more effective and targeted therapies, improving outcomes for patients with GIST.

## 4. Conclusions

This case report of a patient with jejunal GIST perforation highlights the complexities and varied presentations of gastrointestinal stromal tumors. The case underscores the importance of considering GISTs in differential diagnoses, especially in atypical presentations like acute diffuse peritonitis. Our study emphasizes the role of advanced imaging and histopathological analysis, including immunohistochemistry, in accurately diagnosing GISTs. It also illustrates the critical nature of understanding molecular pathways in GISTs for effective management. The successful outcome in this case, post-surgical intervention and careful follow-up, demonstrates the potential for favorable prognosis even in rare and severe cases of GISTs when managed promptly and appropriately.

## Figures and Tables

**Table 1:** Clinical, histopathological, and treatment characteristics of jejunal gastrointestinal stromal tumor (GIST) perforation cases: a comparative review.

Authors	Year	Age	Sex	Clinical Presentation	Location (Distance from Treitz’ Ligament)	Size (cm)	Intraoperative Findings	Mitotic Count (/50 HPF)	Ki67 %	Treatment and Adjuvant Therapy (Duration)	Outcome
Karagülle et al. [13]	2008	70	M	Right-sided abdominal pain, inappetence, nausea, bloating	Jejunum (5 cm)	5	Abscess	0	N/A	Surgical Resection (SR)	13 months ANED
Ku MC et al. [14]	2010	33	F	Acute abdominal pain	Jejunum (multiple tumors)	6.5 × 5.3 × 3.9	Peritonitis	N/A	N/A	SR	N/A
Feng et al. [15]	2011	49	M	Paroxysmal, persistent left abdominal pain	Jejunum (40 cm)	10 × 8	Peritonitis	<5	<5	SR	N/A
Memmi et al. [16]	2012	59	M	Acute abdominal pain	Jejunum (150 cm)	12 × 10 × 9	Peritonitis	7	8	SR	N/A
Shoji et al. [17]	2013	61	M	Sudden abdominal pain, nausea	Jejunum (40 cm)	9 × 7	Peritonitis	0	1	SR + Imatinib (36 months)	36 months ANED
Misawa et al. [18]	2014	70	M	Fever, abdominal pain	Jejunum (near Treitz’s ligament)	10 × 10	Peritonitis	N/A	26	SR + Imatinib	12 months ANED
Alessiani et al. [6]	2014	82	M	Fever, vomiting, diarrhea, diffuse abdominal pain	Jejunum (10 cm)	7 × 5	Peritonitis	16	15	SR + Imatinib	6 months ANDE
Sato et al. [12]	2017	74	M	Vomiting, abdominal pain	Jejunum (100 cm)	14	Peritonitis	N/A	N/A	SR + Imatinib (3 months)	22 months
Meneses et al. [19]	2020	46	M	Left upper quadrant pain, fevers, chills	Jejunum (10 cm)	13 × 6 × 7.5	Peritonitis	<5	10	SR	N/A
Al-Swaiti et al. [4]	2020	59	M	Severe generalized abdominal pain	Jejunum (mild)	11 × 9	Peritonitis	8	N/A	SR + Imatinib	N/A
Our case	2022	76	F	Acute abdominal pain, abdominal distention	Jejunum (150 cm)	35	Peritonitis	>5	25	SR	24 months ANED

Note: ANED—alive with no evidence of disease; SR—surgical resection; HPF—high power field; N/A—not available.

## Data Availability

As a case report and literature review, this article does not include original primary data. The information discussed is sourced from previously published works and the patient’s medical records.
